# Comparative Proteomic Approach Identifies Pkm2 and Cofilin-1 as Potential Diagnostic, Prognostic and Therapeutic Targets for Pulmonary Adenocarcinoma

**DOI:** 10.1371/journal.pone.0027309

**Published:** 2011-11-08

**Authors:** Xing-chen Peng, Feng-ming Gong, Yu-wei Zhao, Liang-xue Zhou, Ying-wei Xie, Hong-li Liao, Hong-jun Lin, Zhi-yong Li, Ming-hai Tang, Ai-ping Tong

**Affiliations:** 1 State Key Laboratory of Biotherapy and Cancer Center, West China Hospital, West China Medical School, Sichuan University, Chengdu, Sichuan, China; 2 Department of Neurosurgery, West China Hospital, Sichuan University, Chengdu, Sichuan, China; 3 Department of Oncology, People's Hospital of Daxian Conuty, Dazhou, Sichuan, China; 4 The First Affiliated Hospital of Wenzhou Medical College, Wenzhou, Zhejiang, China; International Centre for Genetic Engineering and Biotechnology, Italy

## Abstract

Lung cancer is the leading cause of cancer-related death in the world. Non-small cell lung carcinomas (Non-SCLC) account for almost 80% of lung cancers, of which 40% were adenocarcinomas. For a better understanding of the molecular mechanisms behind the development and progression of lung cancer, particularly lung adenocarcinoma, we have used proteomics technology to search for candidate prognostic and therapeutic targets in pulmonary adenocarcinoma. The protein profile changes between human pulmonary adenocarcinoma tissue and paired surrounding normal tissue were analyzed using two-dimensional polyacrylamide gel electrophoresis (2-DE) based approach. Differentially expressed protein-spots were identified with ESI-Q-TOF MS/MS instruments. As a result, thirty two differentially expressed proteins (over 2-fold, p<0.05) were identified in pulmonary adenocarcinoma compared to normal tissues. Among them, two proteins (PKM2 and cofilin-1), significantly up-regulated in adenocarcinoma, were selected for detailed analysis. Immunohistochemical examination indicated that enhanced expression of PKM2 and cofilin-1 were correlated with the severity of epithelial dysplasia, as well as a relatively poor prognosis. Knockdown of PKM2 expression by RNA interference led to a significant suppression of cell growth and induction of apoptosis in pulmonary adenocarcinoma SPC-A1 cells *in vitro*, and tumor growth inhibition *in vivo* xenograft model (P<0.05). In addition, the shRNA expressing plasmid targeting cofilin-1 significantly inhibited tumor metastases and prolonged survival in LL/2 metastatic model. While additional works are needed to elucidate the biological significance and molecular mechanisms of these altered proteins identified in this study, PKM2 and cofilin-1 may serve as potential diagnostic and prognostic biomarkers, as well as therapeutic targets for pulmonary adenocarcinoma.

## Introduction

Lung cancer was a leading cause of cancer-related death and the 5-year overall survival rate was below 16% [1]. According to the WHO's estimation, China will become one of the countries that have a relatively high incidence of lung cancer in the 21st century [2]. Non-small cell lung carcinomas (Non-SCLC) account for almost 80% of lung cancers, of which 40% were adenocarcinomas. Early detection can increase the chances of the patient responding well to treatment, which was evidenced by the effect that 63% of the patients with early stages had an expected five-year survival [3]. Thus, it is no doubt that discovering biomarkers used for the early detection of lung adenocarcinomas and the monitoring of disease progression is very critical. And the identification of novel therapeutic targets would also facilitate drug development for lung adenocarcinomas.

The development of genome-wide screening have displayed that the initiation, development and outcome of lung adenocarcinomas was associated with DNA methylation [4], [5], some genetic mutations, such as K-ras [6], [7], p53 [8] and EGFR [9], as well as altered expression of genes, such as VEGF, S100P and crk [10]. These novel biomarkers have been utilized in the diagnosis, prognosis prediction and treatment of lung cancer. However, mRNA expression does not necessarily correlate with protein level, and posttranslational modifications, such as phosphorylation, can not be predicted from transcription assay result [11]. So it is thought that analysis of the cancer proteome can be more informative than genomics alone [12].

The proteomic approach has resulted in many opportunities and challenges in identifying new tumor markers and therapeutic targets, and in understanding disease pathogenesis [13]. Previous studies have performed the preliminary application of 2-DE in the identification of the biomarkers for lung adenocarcinoma, which has revealed significant changes in the expression levels of a number of proteins, including some metabolic enzymes, signal transduction proteins, oncoproteins and so on [3], [14]. To date, however, few molecular biomarkers for lung adenocarcinoma have been introduced into clinical practice, mainly owing to insufficient validation and the absence of prospective studies. Moreover, few studies have been functionally analyzed for their roles in carcinogenesis, and therefore there is a lack of the fundamental understanding required for clinical applications and a need for a better comprehension of the underlying biological processes [15], [16]. Finally, the previous study population mainly consists of Caucasian while only a few proteomic studies on lung adenocarcinoma of Asian population have been reported [3], [14]. The ethnic differences among patients may contribute to different findings and conclusions.

In the present study, we utilized a 2-DE based proteomic approach to profile the altered proteins between lung adenocarcinoma tissues and corresponding normal lung tissues. Of the identified thirty two dysregulated proteins, two significantly upregulated proteins (PKM2 and cofilin-1) were further studied in the thoughts of their important biological functions. The data presented in this study suggested that both PKM2 and cofilin-1 could be developed as valuable prognostic factors, as well as potential therapeutic targets for lung adenocarcinoma.

## Materials and Methods

### Ethics Statement

Tissue samples and patient data were obtained with informed written consent. The project was approved by the Scientific and Ethical Committee of Sichuan University, China (NO. 090563).

### Tissue specimens

All tissue samples were obtained from West China Hospital, Sichuan University. Samples were examined histologically after staining with H&E staining, and clinicopathologic stage was determined according to the TNM classification system of the International Union against Cancer. Lung adenocarcinoma tissues and corresponding normal tissues were collected from 9 lung adenocarcinoma patients (Table 1). Tumor tissues were incised from the center of tumor mass, and the paired corresponding normal tissues were incised along a surgically called safety border which was verified with frozen section staining. All the tissue specimens were snap-frozen in liquid nitrogen for proteomic analysis. For immunohistochemistry (IHC) analyses, tissue specimens were recruited from the archives of the pathology department of West China hospital. All clinical information was obtained from archives of case history. Informed consent was obtained from all of patients or their relatives for the use of their tissues in the experimental procedures.

**Table 1:** The clinical and pathologic data of patients with lung adenocarcinoma for 2-DE.

Sample No	Gender	Age (year)	Histological type	TNM classification	Clinical stage
1	Male	49	Well diff.	T2bN0M0	II a
2	Male	67	Mod diff.	T2bN1M0	II b
3	Female	52	Poor diff.	T2aN0M0	I b
4	Female	38	Poor diff.	T3N1M0	III a
5	Male	55	Mod diff.	T1N1M0	II a
6	Female	44	Poor diff.	T3N0M0	II b
7	Female	63	Well diff.	T2aN0M0	I b
8	Male	59	Poor diff.	T3N1M0	III a
9	Female	64	Mod diff.	T1N1M0	II a

### 2-DE and image analysis

Tissues were ground into powder in liquid nitrogen and lysed in lysis buffer (7 M urea, 2 M thiourea, 4% CHAPS, 100 mM DTT, 0.2% pH 3–10 ampholyte, Bio-Rad, USA) containing protease inhibitor cocktail 8340 (Sigma, St. Louis, MO). After centrifugation followed by vortex and incubation, supernatant was precipitated with cold actone. The pellet was redissolved in the lysis buffer. The protein concentrations were determined using DC protein assay kit (Bio-Rad). Protein samples (2 mg) were applied to IPG strip (17 cm, pH 3–10 NL, Bio-Rad) using a passive rehydration method. After 12–16 h of rehydration, the strips were transferred to an IEF Cell (Bio- Rad). IEF was performed as follows: 250 V for 30 min, linear; 1000 V for 1 h, rapid; linear ramping to 10 000 V for 5 h and finally 10 000 V for 4 h. Second dimension was performed using 12% SDS-PAGE at 30 mA constant current per gel after equilibration. Gels were stained using CBB R-250 (Merck, Germany) and scanned with a Bio-Rad GS-800 scanner. Pooled samples were prepared by mixing the 9 clinical samples with equal amount. Three independent runs were made for the pooled samples to ensure the accuracy of analyses. The maps were analyzed by PDQuest software Version 6.1 (Bio-Rad). The quantity of each spot in a gel was normalized as a percentage of the total quantity of all spots in that gel and evaluated in terms of OD. For statistical analysis, paired t-test was performed to compare data from the three repeated experiments. Only spots that showed consistent and significant differences (±over twofold, p<0.05) were selected for analysis with MS.

### In-gel digestion

In-gel digestion of proteins was carried out using MS-grade Trypsin Gold (Promega, Madison, WI) according to the manufacturer's instructions. Briefly, spots were cut out of the gel (1–2 mm diameter) using a razor blade, and destained twice with 100 mM NH_4_HCO_3_/50% ACN at 37°C for 45 min in each treatment. After drying, the gels were preincubated in 10–20 µl trypsin solution for 1 h. Then, 15 µl digestion buffer was added (40 mM NH_4_HCO_3_/10% ACN) to cover each gel and incubated overnight at 37°C. Tryptic digests were extracted using MilliQ water initially, followed by two times extraction with 50% ACN/5% TFA for 1 h each time. The combined extracts were dried in a vacuum concentrator at room temperature. The samples were then subjected to MS analysis.

### ESI-Q-TOF analysis and protein identification

Mass spectra were acquired using a Q-TOF mass spectrometer (Micromass, Manchester, UK) fitted with an ESI source (Waters). Tryptic digests were dissolved in 18 µl 50% ACN. For ESI-Q-TOF analysis, the automatic scan rate was 1.0 s with an inter-scan delay of 0.02 s, and the system was operated at 3.0 kV. Spectra were accumulated until a satisfactory S/N had been obtained. Parent mass peaks with the range from 400 to 1600 m/z were picked out for MS/MS analysis. The collision energy was chosen to vary between 18 and 57 eV depending on the mass of the precursor. The MS/MS data were acquired and processed using MassLynx V 4.1 software (Micromass) and were converted to PKL files by the ProteinLynx 2.2.5 software (Waters). The PKL files were analyzed using the MASCOT search engine (http://www.matrixscience.com). Database searches were carried out using the following parameters: Database, Swiss-Prot; taxonomy, homo sapiens; enzyme, trypsin; mass tolerance, ±0.1 Da; MS/MS tolerance, ±0.05 Da; and an allowance of one missed cleavage. Fixed modifications of cysteine carboamidomethylation, and variable modifications of methionine oxidation were allowed. The data format was selected as Micromass PKL and the instrument was selected as ESI-Q-TOF. Proteins with probability based MOWSE scores, derived from ions scores as a non-probabilistic basis for ranking protein hits when using Mascot searching engine, exceeding their threshold (p<0.05), and with the molecular weight and pI consistent with the gel regions from which the spots were excised, were considered to be positively identified.

### Western blotting

Proteins were extracted in RIPA buffer (50 mM Tris-base, 1.0 mM EDTA, 150 mM NaCl, 0.1% SDS, 1% Triton X-100, 1% Sodium deoxycholate, 1 mM PMSF) and quantified by the DC protein assay kit (Bio-Rad). Samples were separated by 12% SDS-PAGE and transferred to PVDF membranes (Amersham Biosciences). After blocking with 5% non-fat milk in Tris-buffered saline (TBS), 0.1% Tween 20 for 1 h, membranes were incubated overnight at 4°C with respective primary antibodies. Specific primary antibodies performed included rabbit anti-human cofilin-1 antibody (Abcam, Cambridge, UK), and rabbit anti-human PKM2 antibody (Santa Cruz Biotechnology, Santa Cruz, CA). After that, the blots were incubated with secondary antibody conjugated to HRP for 2 h at room temperature. Target proteins were detected by enhanced chemiluminescence reagents (Amersham Pharmacia Biotech, Piscataway, USA). A monoclonal anti-β-actin antibody (Sigma) was used as a loading control.

### Immunohistochemistry and Correlation Analysis

Sections were stained by Envision System-HRP method (DakoCytomation Inc, Carpinteria, CA), according to kit manufacturer's instructions. Specific antibodies performed included rabbit anti-human cofilin-1 antibody(Abcam, Cambridge, UK), rabbit anti-human PKM2 antibody (Santa Cruz Biotechnology, Santa Cruz, CA) and rabbit anti-human PCNA antibody (Santa Cruz Biotechnology, Santa Cruz, CA). Saturation and intensity of immunostained cells was evaluated over 8 visual fields at a power of ×400 under a light microscope (Olympus Optical, Tokyo, Japan). In statistical analysis, with reference to Jeffrey's study [17], total staining of PKM2 or cofilin-1 were scored as the product of the staining intensity (on a scale of 0–3: negative = 0, weak = 1, moderate = 2, strong = 3) × the percentage of cells stained (positively recorded on an ordered categorical scale: 0  =  zero, 1 = 1–25%, 2  =  26–50%, 3 = 51–100%), which resulted in a scale of 0–9. The evaluation was performed by two independent investigators, without any prior knowledge of each patient's clinical information and outcome. Any discrepancy between the two evaluators was resolved by reevaluation and careful discussion until agreement was reached. In correlation analysis, the survival data of patients were classified as weak (0–3), moderate (3.1–6), or strong (6.1–9) staining of PKM2 or cofilin-1 or both biomarkers. Multivariate analyses were carried out using Cox proportional hazard model.

### Short hairpin RNA construction and production

ShRNA with the sequence 5′-CCGGGCTGTGGCTCTAGACACTAAACTCGAGTTTAGTGTCTAGAGCCACAGCTTTTTG-3′was used to inhibit the expression of PKM2 as described previously [18]. According to the previous study [19], the shRNA with the sequence 5′-GGATCCCGAGCGGACATTTAGGAACTTTCAAGAGAAGTTCCTAAATGTCCGCTCTTTTTTCCAAAAGCTTT-3′was used to inhibit the expression of cofilin-1. HK sequence, which has no homology with any mammalian sequence, was used as negative control. Eukaryotic expression vector pGenesil-2 (Genesil Biotechnology, Wuhan, China) was used to construct the shRNA expressing plasmid. Plasmids were extracted using a Qiagen Plasmid Mega Kit (Qiagen, Hilden, Germany) and stored at −20°C.

### Liposome preparation

DOTAP:chol liposome was prepared using the procedure described previously [20]. Briefly, cationic lipid DOTAP was mixed with neutral lipid Chol at equimolar concentrations. Mixed lipids were dissolved in chloroform in a 100 ml-round-bottomed flask. Then, clear solution was rotated on a Buchi rotary evaporator at 30°C for 30 min to make a thin film, and the flask containing thin lipid film was dried under vacuum for 15 min. The film was hydrated in 5% dextrose in water (D5W) to give a final concentration of 7 mM DOTAP and 7 mM chol, referred to as 7 mM DOTAP:chol. The hydrated lipid film was rotated in a water bath at 50°C for 45 min and then 35°C for 10 min. The mixture was allowed to stand in the parafilm-covered flask at room temperature overnight, after which the mixture was sonicated at low frequency for 5 min at 50°C, transferred to a tube, and heated for 10 min at 50°C. The mixture was sequentially extruded through Millipore (Billerica, MA) polycarbonate membrane of decreasing size: 0.2 µm for 5 times and 0.1 µm for 3 times using syringes. Liposomes were stored under argon gas at 4°C. DOTAP was purchased from Avanti Polar Lipids (Alabaster, AL), and highly purified Chol was purchased from Sigma (St. Louis, MO).

### Cell culture and transfection

Human lung adenocarcinoma cells SPC-A1 and murine Lewis lung carcinoma cell line LL/2 were obtained from American Type Culture Collection. Cells were maintained in RPMI 1640 or DMEM (Life Technologies) containing 10% FBS (Life Technologies), 100 units/ml penicillin (Thermo) and 100 units/ml Streptomycin (Thermo). Cells were grown in a 5% CO2 incubator at 37°C.

Cells were grown on a 6-well plate in growth medium until they reached 70% confluence. Liposomes and plasmids were diluted in antibiotics and serum-free media, respectively, and then combined at a ratio of 2.5∶1(5 µg liposome/2 µg DNA). The combinations were transfected to the cells according to previous studies [20].

### The quantitative assessment of apoptosis

Harvested cells were stained with 1 ml hypotonic fluorochrome solution containing 50 µg/ml propidium iodide in 0.1% sodium citrate plus 0.1% Triton X-100. Then flow cytometric analysis were performed to identify apoptotic cells and to measure the percentage of sub-G1 cells using a flow cytometer (ESP Elite; Coulter).

### Colony Formation assay

Cells transfected with ShRNA complexed with liposome were seeded at 2×10^2^ per well and allowed to grow for an additional 14 days. The colonies were then fixed with methanol and stained with Crystal Violet (Sigma, St Louis, MO) and counted under a microscope.

### Therapy in SPC-A1 xenograft tumor model

Female nude *BALB/c* mice of 6 to 8 weeks old were purchased from experimental animal center of Sichuan University (Chengdu, Sichuan province, China) and were housed in our animal research facility. Mice were kept in groups of five per cage and fed with clean food and water. Animals were acclimatized for 1 week before use and maintained throughout at standard conditions: 24±2°C temperature, 50±10% relative humidity.

Female *BALB/C* nude mice were challenged subcutaneously with 5 × 10^6^ SPC-A1 tumor cells per mouse in right flank. After 8 days, when the tumor diameters were about 0.6–0.8 cm, the tumor-bearing mice were randomly assigned into the following four groups and each mouse received the corresponding treatment by caudal vein injection: (a) PBS group, 100 µl of PBS; (b) Lipo group, liposome 25 µg (volume = 100 µl); (c) Negative control (NC group), pGenesil-2-HK-shRNA 10 µg complexed with liposome 25 µg (volume = 100 µl); (d) ShRNA group, pGenesil-2-PKM2-shRNA 10 µg complexed with liposome 25 µg (volume = 100 µl). Injections were performed every three days, and tumor volumes were evaluated according to the following formula: tumor volume (mm^3^)  =  0.52 × length × width^2^. Side effects of treatment and the weight, appetite, and behavior of the mice were recorded for 27 days, after which the mice were sacrificed.

Tumor net weight of each mouse was measured. The dissected tumors were fixed in neutral buffered formalin and embedded in paraffin, and sections (5 µm) were used for histologic analysis.

### TUNEL assay

Presence of apoptotic cells within tumor sections was evaluated by TUNEL (terminal deoxynucleotidyltransferase-mediated dUTP nick-end labeling) technique using the DeadEnd Fluorometric TUNEL System (Promega, Madison, WI) following the manufacturer's protocol. Percent apoptosis was determined by counting the number of apoptotic cells and divided by the total number of cells in the field (5 high power fields/slide).

### Therapy in lung metastatic model

Female C57BL/6 mice (6–8 weeks old) were purchased from experimental animal center of Sichuan University (Chengdu, Sichuan province, China) and were housed in our animal research facility. Each mouse was inoculated with LL/2 cells (5 × 10^5^) via the tail vein to establish lung metastatic model. These lung metastatic mice were randomly assigned into the following four groups at day 12 and each mouse received the corresponding treatment by caudal vein injection: (a) PBS group, 100 µl of PBS; (b) Lipo group, liposome 25 µg (volume = 100 µl); (c) Negative control (NC group), pGenesil-2-HK-shRNA 10 µg complexed with liposome 25 µg (volume = 100 µl); (d) ShRNA group, pGenesil-2-cofilin-1-shRNA 10 µg complexed with liposome 25 µg (volume = 100 µl). Caudal vein injections were performed every three days. After 6 mice from each group were sacrificed at day 30, lung net weight of each mouse was measured. Autopsy was performed to determine the number of the metastatic nodules of lung. The other mice (eight mice/group) were followed for survival time.

### Toxicity assessment

Normal human bronchial epithelial cells BEAS-2B were obtained from American Type Culture Collection. Colony Formation assays were conducted as mentioned above. We also conducted an in vivo study of the effects of PKM2 or cofilin-1-ShRNA on the lung weight of tumor non-bearing mice to check for non-specific effects. Tumor non-bearing mice were randomly assigned into the following four groups and each mouse received the corresponding treatment by caudal vein injection: (a) PBS group, 100 µl of PBS; (b) Lipo group, liposome 25 µg (volume = 100 µl); (c) Negative control (NC group), pGenesil-2-HK-shRNA 10 µg complexed with liposome 25 µg (volume = 100 µl); (d) PKM2 or cofilin-1-ShRNA group, shRNA 10 µg complexed with liposome 25 µg (volume = 100 µl). When treatments were finished, mice were sacrificed, and lung were harvested, weighed and fixed in 4% formaldehyde solution. Tissues were then sectioned, stained with H&E, and observed by two pathologists in a blinded manner.

### Statistical analysis

Paired t-test and one way ANOVA was used to analyze differences between groups. Survival curves were generated according to the Kaplan-Meier method and the statistical analyses were performed using Log-rank test. Relevance analysis of ordinal data was performed by cross X^2^ test. P<0.05 was considered significant in all analyses.

## Results

### 2-DE and ESI-Q-TOF-MS/MS analysis

**Figure 1:**
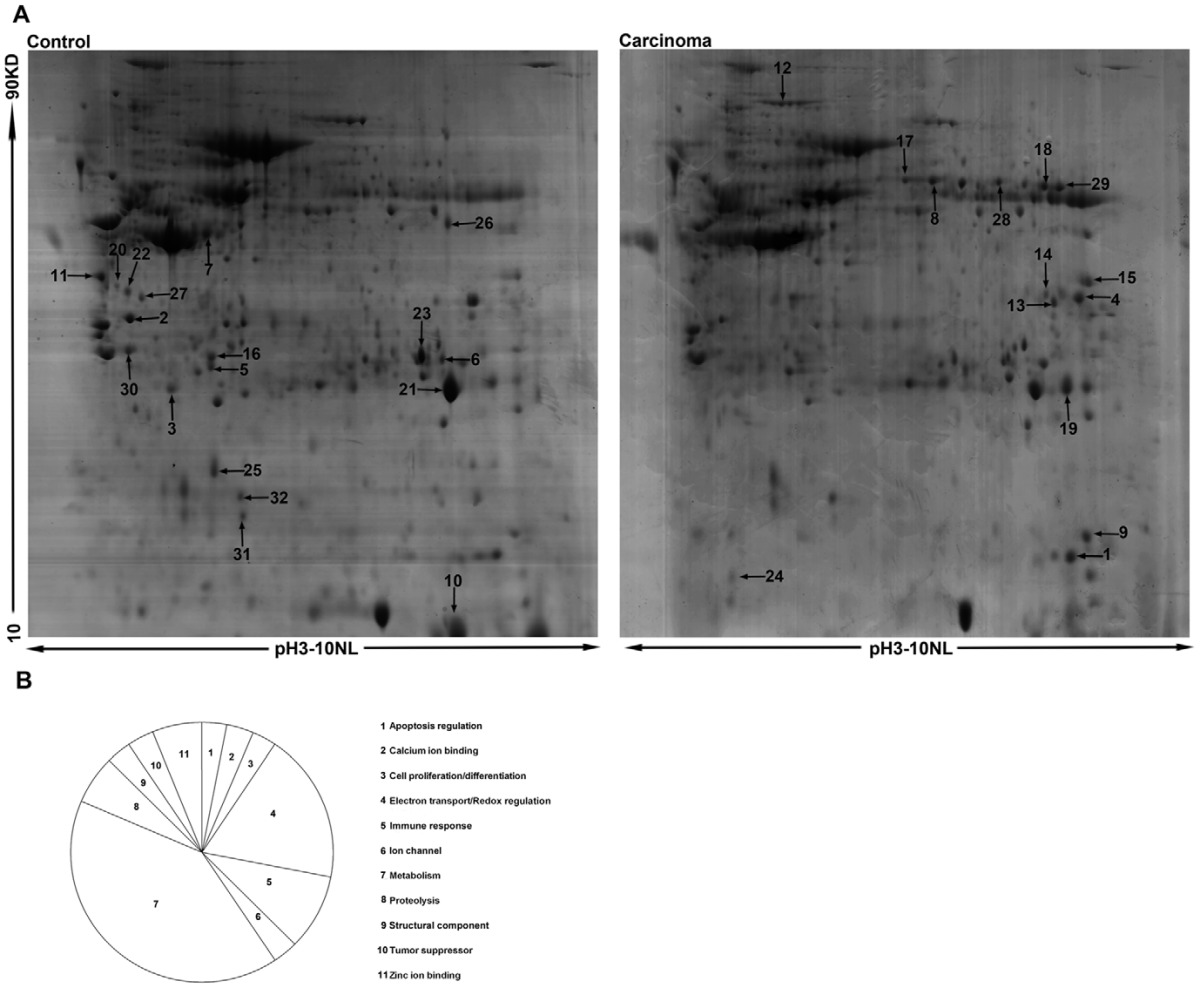
Protein profiles between lung adenocarcinoma tissues and corresponding normal lung tissues. (**A**)**.** Representative 2-DE maps of lung adenocarcinoma and normal tissues, with 2 mg protein loading amount and visualized by Coomassie blue staining. Arrows indicate identified protein spots significantly and consistently altered between carcinoma tissue and control normal tissues. (**B**)**.** Cluster analysis, the altered proteins were involved in diverse biological processes, including metabolism (40.6%), electron transport/redox regulation (18.8%), immune response (9.4%), proteolysis (6.3%), and so on.

**Figure 2:**
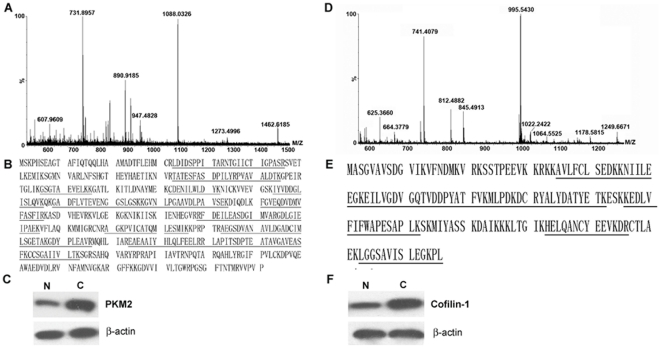
ESI-Q-TOF MS/MS identification and western blot validation of representative proteins PKM2 and cofilin-1. (**A & D**)**.** Mass spectrogram of tryptic peptides of PKM2 and cofilin-1. (**B & E**). Protein sequence of PKM2 and cofilin-1. The matched peptides are underlined. (**C & F**). Western blot validation of PKM2 and cofilin-1 with pooled clinical samples. N: normal tissues; C: lung adenocarcinoma tissue.

2-DE analysis for pooled tumor and normal tissues from 9 patients (Table 1) were independently repeated for three times. A pair of representative 2-DE maps was shown in Fig. 1A. A total of 32 unique proteins were positively identified by ESI-Q-TOF-MS/MS (arrows in Fig. 1A and listed in Table S1). Cluster Analysis revealed that the altered proteins were involved in diverse biological processes, including metabolism (40.6%), electron transport/redox regulation (18.8%), immune response (9.4%) and so on (Fig. 1B). Among them, both PKM2 and cofilin-1 were identified with significant alterations. PKM2 was up-regulated 2.9-fold and cofilin-1 was up-regulated 3.7-fold in tumor compared to paired surrounding normal tissue (p<0.05). The two proteins were selected as examples showing ESI-Q-TOF-MS/MS analysis (Fig. 2). As shown, PKM2 was positively identified with 18 matched peptides and a MOWSE score of 250 and cofilin-1 with 15 matched peptides and a MOWSE score of 280.

### Western blot validation and IHC analysis

**Figure 3:**
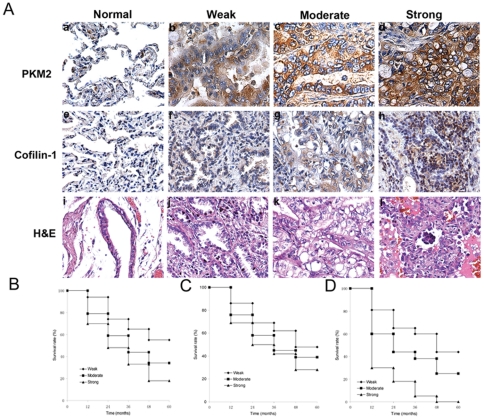
Immunohistochemical (IHC) analysis. (**A**)**.** IHC staining against PKM2 and cofilin-1. (**B**)**.** Kaplan-Meier survival curves showed the correlation between higher levels of PKM2 expression and lower survival rates (p<0.05). (**C**)**.** Kaplan-Meier survival curves showed the correlation between higher levels of cofilin-1 expression and lower survival rates (p<0.05). (**D**)**.** Kaplan-Meier survival curves showed the combination of PKM2 and cofilin-1 as predictor correlated more closely with prognosis of lung adenocarcinoma (p<0.05).

As shown in Fig. 2 C, F, overexpression of PKM2 or cofilin-1 was observed in pooled carcinoma samples compared with corresponding adjacent normal samples when subjected to western blot assays, which was consistent with the observation made in 2-DE analysis. To further study the potential oncogenic properties of PKM2 and cofilin-1, and to assess their potential prognostic and therapeutic value, IHC was performed to examine PKM2 and cofilin-1 expression in 98 paraffin-embedded tissues. As shown in Table 2 and Fig. 3 A, of the 30 adjacent normal tissues, positive staining of PKM2 was rarely detected and total staining score was only 0.80±1.47. However, the other three groups, including well differentiated, moderately differentiated and poorly differentiated, showed a remarkable increasing trend of positive staining of PKM2, with 1.86±1.56, 3.41±2.40, 7.18±1.65 total staining score, respectively. Moreover, similar trend was found in IHC staining of cofilin-1 (Table 3 and Fig. 3 A). These result indicated that increased expression of PKM2 and cofilin-1 were paralleled with increasing severity of epithelial dysplasia (Fig. 3 A).

**Table 2:** PKM2 immunostaining results of patients with lung adenocarcinoma.

Tissue type	Number	Male	Female	Positive rate	Intensity	Total staining Score^a,^
Adjacent normal tissues	30	14	16	0.47±0.68	0.57±0.86	0.80±1.47
Well	21	11	10	1.43±0.87	1.05±0.59	1.86±1.56
Moderately	37	16	21	1.73±0.96	1.76±0.93	3.41±2.40
Poorly	40	19	21	2.75±0.44	2.63±0.49	7.18±1.65

a: Total staining of PKM2 was scored as the product of the staining intensity (on a scale of 0–3) × the percentage of cells stained (on a scale of 0–3).

**Table 3:** Cofilin-1 immunostaining results of patients with lung adenocarcinoma.

Tissue type	Number	Male	Female	Positive rate	Intensity	Staining Score^a,^
Adjacent normal tissues	30	14	16	0.53±0.73	0.50±0.68	0.67±0.99
Well	21	11	10	0.90±0.77	0.86±0.73	1.14±1.06
Moderately	37	16	21	1.21±0.79	1.65±1.01	2.58±2.26
Poorly	40	19	21	2.53±0.64	2.55±0.55	6.43±2.12

a: Total staining of cofilin-1 was scored as the product of the staining intensity (on a scale of 0–3) × the percentage of cells stained (on a scale of 0–3).

To assess the correlation between overexpression of PKM2 or cofilin-1 and patient survival rate, 98 patients were retrospectively studied. As shown, when PKM2 was assessed as a single marker, the five year survival rates were 55%, 34%, 18% for weak, moderate and strong staining samples, respectively (Fig. 3 B). And the five year survival rates were 48%, 39%, 28% for weak, moderate and strong staining samples, respectively, when cofilin-1 was assessed as a single marker (Fig. 3 C). Multivariate analyses using Cox proportional hazard model showed that both PKM2 and cofilin-1 could be developed as an independent prognostic factor for lung adenocarcinoma. At last, the combination of PKM2 and cofilin-1 as predictors for survival were also tested. The 5-year overall survival rate for the PKM2-/cofilin-1- strongly positive group was very poor (0%), compared with the other two groups (Fig. 3 D), suggesting combination of the two markers may facilitate a more accurate prognosis of lung adenocarcinoma.

### Clonogenic formation assay and apoptosis assay

**Figure 4:**
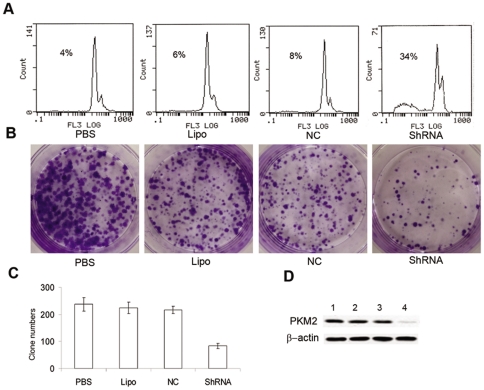
Suppression of PKM2 showed significantly antitumor effects. (**A**)**.** Flow cytometry assay. SPC-A1 cells treated with PKM2-ShRNA plasmids for 48 h have a significantly sub-G1 peak compared with control groups. (**B**)**.** Colony formation assay. Following transfection, SPC-A1 cells were allowed to grow for additional 14 days. The colonies were then fixed with methanol and stained with Crystal Violet. (**C**)**.** Clonogenic formation assay showed that the clone numbers were 237±24.78, 224±21.61, 216±14.09, and 83±9.43 in PBS group, Lipo group, NC group, and PKM2-ShRNA treated group, respectively. Inhibition ratio of PKM2-ShRNA group was 64.9% compared with PBS group (Dunnett-t test, P<0.01). (**D**)**.** Expression of PKM2 in SPC-A1 was significantly down-regulated by PKM2-ShRNA plasmids. (1) PBS group; (2) Lipo group; (3) NC group; (4) PKM2-ShRNA group.

To study the potential tumorigenesis function of PKM2, human lung adenocarcinoma cell line SPC-A1 was treated with PKM2-ShRNA. As shown in Fig. 4A, SPC-A1 cells have an obvious sub-G1 peak (34%) compared with control groups after treatment with PKM2-ShRNA plasmids for 48 h by flow cytometry assay. Clonogenic formation assays demonstrated that upon 14-day continuous culture, the clone numbers were 237±24.78, 224±21.61, and 216±14.09 in PBS group, Lipo group, and NC group, respectively (Fig. 4B, C). Meanwhile the clone number in the PKM2-ShRNA treated group was 83±9.43 with an inhibition ratio of 64.9% (Dunnett's t test, p<0.01) (Fig. 4B, C). Expression of PKM2 in SPC-A1 was significantly down-regulated by PKM2-ShRNA transfection (Fig. 4D).

### Anti-tumor effects of PKM2-ShRNA plasmids in vivo

**Figure 5:**
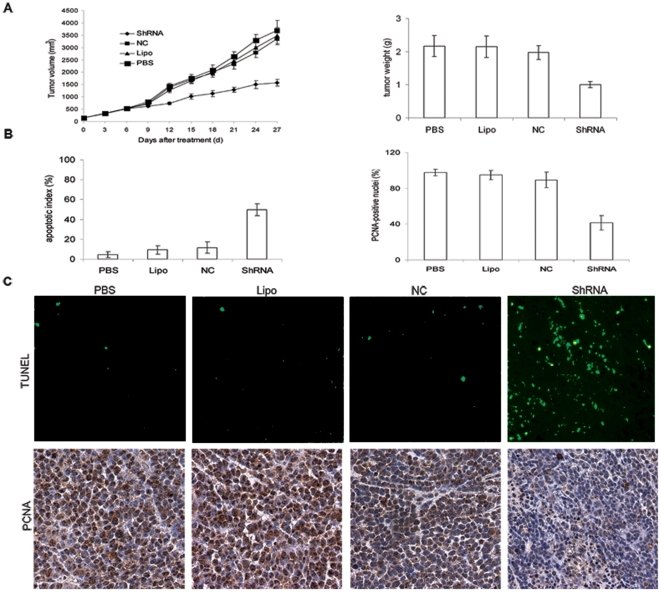
Suppression of PKM2 showed antitumor effects in xenograft mice model. (**A**)**.** Tumor volume growth curve and tumor weight after caudal vein injection of PKM2-ShRNA plasmids complexed with cationic liposome (DOTAP/Chol). PKM2-ShRNA treatment resulted in significantly decreased tumor growth, compared with control groups (P<0.05). (**B & C**)**.** TUNEL assay and PCNA IHC staining showed the average percentage of PCNA- and TUNEL-positive-staining nuclei (counted in five random fields) were significantly lower (PCNA assay) or higher (TUNEL assay) compaired with control groups (P<0.01).

As shown in Fig 5A, treatment with PKM2-ShRNA plasmids resulted in primary tumor growth regression of 57.3%, 54.7% and 52.9% when compared with PBS, Lipo and NC groups respectively (P<0.05). The average weight of the tumors in PKM2-ShRNA plasmids treated group was reduced by 54%, 53.5% and 49.4% when compared with PBS, Lipo and NC groups respectively (P<0.05). To obtain additional insight into the in vivo effects, tumor cell apoptosis and proliferation were assessed by TUNEL assay and PCNA immunoreactivity analysis. Fig.5 B & C showed a significantly greater percentage of TUNEL-positive nuclei in PKM2-ShRNA group than control groups treated with PBS, Lipo, or NC (49.9±6.07 versus 4.5±2.76, 9.3±4.37, or 11.5±5.74, Dunnett-t test, P<0.01). In contrast, percentages of PCNA-positive nuclei in control groups reached 97.8±3.62, 94.9±5.06, 89.3±8.64 for PBS, Lipo, and NC group respectively, whereas corresponding values for PKM2-ShRNA-treated group reached only 41.5±7.91, which was on average over 57% smaller than PBS control (Student's t test, P<0.01).

### Cofilin-1 silencing Reduced metastatic nodules and prolonged survival in pulmonary metastatic mouse model

**Figure 6:**
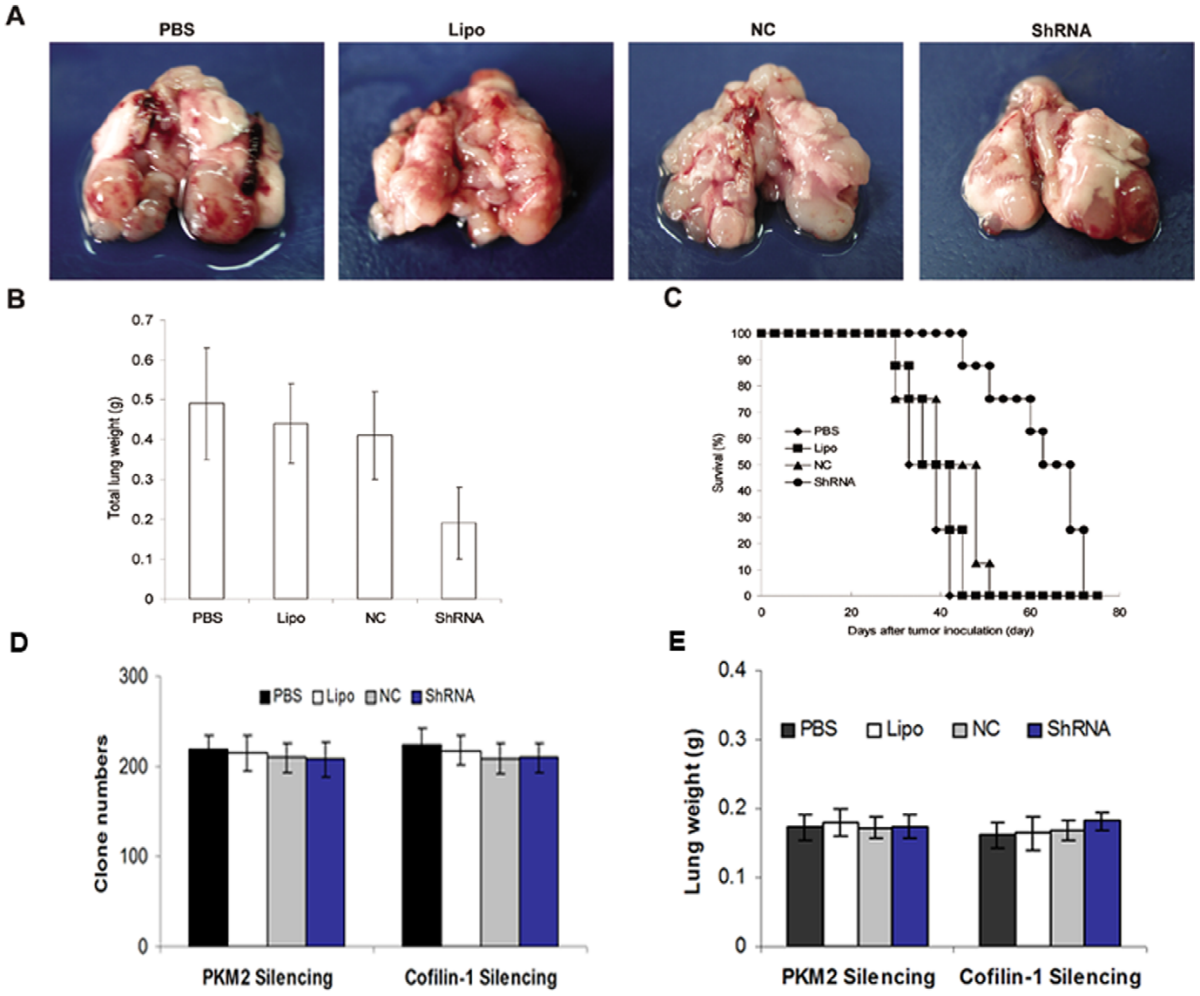
Cofilin-1 suppression retarded pulmonary metastatic tumor growth and enhanced mice survival. (**A**)**.** Representative photos of metastatic-tumor-bearing lung tissues (**B**)**.** Lung weight changes of mice treated with cofilin-1-ShRNA (P<0.05). (**C**)**.** Kaplan-Meier survival curves of tumor-bearing mice (Log-rank test, P<0.01). (D). Treatment with PKM2 or cofilin-1-ShRNA did not affect the growth of normal human bronchial epithelial cells BEAS-2B by clonogenic formation assays (P>0.05). (E). In vivo studies showed that the lung weight of PKM2 or cofilin-1-ShRNA treated group (normal mice, i.e. not bearing tumor) did not have significant changes compared with the control groups (P>0.05).

The in vivo antitumor effects of cofilin-1-ShRNA complexed with cationic liposome (DOTAP/Chol) were evaluated in terms of pulmonary metastatic tumor growth and survival in mice. As shown in Figure 6 A, the tumor nodules were significantly reduced in cofilin-1-ShRNA -treated mice. The average lung weight of mice treated with cofilin-1-ShRNA was 61.2%, 56.8% and 53.7% less than that treated with PBS, Lipo and NC respectively (P<0.05, Figure 6 B). More importantly, treatment of cofilin-1-ShRNA exhibited a significant prolongation of survival compared to control mice (P<0.01, Figure 6 C).

### Toxicity assessment

Treatment with PKM2 or cofilin-1-ShRNA did not affect the growth of normal human bronchial epithelial cells BEAS-2B by clonogenic formation assay (P>0.05, Figure 6 D). In vivo studies also showed that the lung weight from PKM2 or cofilin-1-ShRNA treated group (normal mice, i.e. not bearing tumor) did not have significant changes compared with control groups (P>0.05, Figure 6 E). Furthermore, no toxic pathological changes in lung were found via microscopic examination (data not shown).

## Discussion

As one of the most common malignancies worldwide, lung adenocarcinoma remains a major health problem with increasing incidence rates even to date [1], [21]. Thus, there is an urgent need to identify novel molecular targets for diagnosis, prognosis and treatment of lung adenocarcinoma. In the present study, the global protein profiles were compared between lung adenocarcinoma tissues and corresponding normal lung tissues using a 2-DE and MS/MS-based approach. A total of 32 differentially expressed proteins were identified and cluster analysis revealed that these altered proteins were involved in diverse biological processes, mainly including metabolism (40.6%), electron transport/redox regulation (18.8%), immune response (9.4%), and proteolysis (6.3%).

Among these identified proteins, NME1 gene, also called Nm23, is the first of 13 identified tumor metastasis suppressor genes [22], [23]. NME1 was shown to be decreased 2.2-fold in lung adenocarcinoma in the present study. NME1, a member of the nucleoside diphosphate kinase family of proteins, possesses multiple biochemical functions, including interactions with numerous proteins with nucleoside diphosphate kinase activity and histidine protein kinase activity [24]. The overexpression of NME1 contributes to reduced anchorage-independent colonization, inhibited invasion and motility in response to multiple factors, and up-regulated differentiation in *in vitro* assays [25], [26]. Decreased NME1 protein in tumor samples is correlated with characteristics of aggressive cancer, such as poor clinical survival and prognosis, lymph node infiltration, as well as invasiveness and metastasis in a variety of tumor types, including breast, lung, melanoma, gastric, ovarian, cervical, and hepatocellular carcinomas [27]. Another interesting altered gene was fumarate hydratase (FH), a key enzyme in tricarboxylic acid cycle. It had been reported that individuals with loss of FH activity are at risk for the development of leiomyomas of the skin and uterus (fibroids) as well as kidney cancer [28], [29], [30]. Genetic analysis of tumor samples indicates that FH acts as a tumor suppressor gene [30]. FH was shown to be decreased 3.4-fold in lung adenocarcinoma in the present study.

There are several dramatically changed proteins (N/A, Table S1) which were potentially related to lung cancer genesis. For example, apolipoprotein A-1 was found downregulated in pulmonary adenocarcinoma by 2-DE coupled to MALDI-TOF peptide mass fingerprinting and was presently recognized as a new biomarker for pulmonary adenocarcinoma [31]. Overexpression of Voltage-dependent anion-selective channel protein 1 (VDAC1) predicts shorter time to recurrence and overall survival for non-small cell lung cancer [32]. On the other hand, tumor tissue is composed of both cancer cells and stromal cells recruited from normal tissue, such as fibroblastic cells, endothelial cells, and cells of hematopoietic origin. At the late-stage, the interactions between stromal cells and tumor cells often result in histologic vascular thrombosis and tumor necrosis. So some of the factors identified as differentially expressed in the present study were not directly related to tumor cell development. And some of these proteins were also reported by other proteomic studies previously. For example, fibrinogen gamma were found to be over-expressed in pancreatic cancer [33], some coagulation factors were reported to be candidate cancer biomarkers [34], and coagulation factor VII even can be secreted by cancer cell themselves [35]. Due to post-translational modification, isoform, amino acid composition, hydrophobicity, three dimensional structure and so on, in 2-DE, it happens very often that several adjacent spots were identified by MS to be the same protein. For instance, spot 12 in figure 1 is likely to be a phosphorylated isoform of fibrinogen gamma [33], [36]. Further study of the molecular mechanism of these changed proteins in cancer genesis will help us find new diagnostic and therapeutic approaches for lung cancer.

Pyruvate kinase (PK), one of the key glycolytic enzymes, has four isoforms in mammals, M1, M2, L, and R that are differentially expressed in different cell types [37]. The original tissue specific pyruvate kinase (i.e., type L, R, and M1) is replaced by PKM2 during tumorigenesis. Previously studies had implied that this change could help tumor cells to produce lactic acid through glycolysis, rather than to produce energy through mitochondrial oxidative phosphorylation, and help tumor cells to survive in low glucose and low oxygen environments and facilitate tumor invasion [38], [39]. PKM2 upregulation has been observed in numerous cancers including lung, gastric, cervical, colorectal cancers [38], [40]-[42]. Increasing studies has also shown that inhibition of glycolysis signaling is a very promising approach for cancer treatment [38], [43]. However, no study has been performed previously to assess if there were any correlations among PKM2 expression, histo-differentiation, and survival rate of lung adenocarcinoma and to examine if PKM2 can be used as an effective target for lung cancer therapy. Our study showed that overexpression of PKM2 was correlated with a low degree of differentiation, and survival analysis also displayed PKM2 could be developed as an independent prognostic factor for lung adenocarcinoma. And consistent with previous studies [18], [38], [44], our results also showed that PKM2 silence alone can retard tumor cell growth by inducing apoptosis and inhibiting proliferation both in vitro and in vivo. Obviously, more extensive investigations are required to elucidate the complex molecular functions of PKM2 in lung adenocarcinoma.

Cofilin-1 is one of the main proteins in charge of cell motility that is regulated by many factors such as phosphorylation, pH, binding of phosphoinositides, and subcellular compartmentalization. Local activation of cofilin-1 by uncaging induces lamellipod formation and sets the direction of cell motility. The overexpression of cofilin can increase the velocity of cell migration in dictyostelium [45] and in human glioblastoma cells [46]. The spontaneous overexpression of cofilin has been detected in the invasive subpopulation of tumor cells in mammary tumors [47]. In the present study, cofilin-1 was found to be overexpessed in lung adenocarcinoma and overexpression of cofilin-1 was correlated with a low degree of differentiation. Furthermore, Kaplan-Meier survival analysis showed that cofilin-1 could serve as an independent prognostic factor for lung adenocarcinoma.

Moreover, the results from the combination of PKM2 and cofilin-1 as predictor for survival showed that the 5-year overall survival rate for the PKM2-/cofilin-1- strongly positive group was very poor (0%), compared with the other two groups, which showed a significantly better outcome. It has been accepted that single marker will not have the sensitivity and specificity necessary to be used on its own for diagnosis/prognosis of tumors. The multiparametric approach is likely to improve the sensitivity and specificity.

Considering the biological functions of cofilin-1 in cell motility, we evaluated the effects of cofilin-1 silencing in lung cancer metastases in vivo. Our results showed that cofilin-1-ShRNA delayed tumor metastasis and prolonged mice survival obviously, which suggested that cofilin-1 might serve as a potential target for lung metastases therapy.

In conclusion, in the present study, we have found thirty two differentially expressed proteins in human pulmonary adenocarcinoma using 2-DE-MS/MS proteomic methods. Pathological analysis showed that PKM2 and cofilin-1 are promising diagnostic and prognostic biomarkers for lung adenocarcinoma. Further in vitro and in vivo analysis indicated that silencing of PKM2 or cofilin-1 showed significantly antitumor effects, suggesting that both of them may serve as potential therapeutic targets for pulmonary adenocarcinoma.

## Supporting Information

Table S1 **Identified proteins by MS/MS analysis.**
(DOC)
