# Supplementary material for: Comparative Proteomic Approach Identifies Pkm2 and Cofilin-1 as Potential Diagnostic, Prognostic and Therapeutic Targets for Pulmonary Adenocarcinoma
Source: PLoS One. 2011 Nov 8;6(11):e27309. doi: 10.1371/journal.pone.0027309 (PMC3210781; doi:10.1371/journal.pone.0027309)
Supplement: Table S1 — Identified proteins by MS/MS analysis. (DOC) [file pone.0027309.s001.doc]

Table S1 Identified proteins by MS/MS analysis

| Spot No. | Protein description | Gene name | Function | Accession No. | Theoretical Mr/pI ^a^ | Score | No. of pep ^b^ | Fold change ^c^ |
| --- | --- | --- | --- | --- | --- | --- | --- | --- |
| 1 | Peptidyl-prolyl cis-trans isomerase A | PPIA | Metabolism | P62937 | 17881/7.82 | 214 | 13 | ↑2.3±0.4 |
| 2 | Annexin A5 | ANXA5 | [Calcium ion binding](http://www.ebi.ac.uk/ego/DisplayGoTerm?id=GO:0005509) | P08758 | 35937/4.94 | 1145 | 42 | ↓3.2±0.7 |
| 3 | Apolipoprotein A-I | APOA1 | Metabolism | P02647 | 28078/5.27 | 315 | 21 | ↓N/A |
| 4 | Ig gamma-1 chain C region | IGHG1 | immune response | P01857 | 36106/9.31 | 84 | 11 | ↑2.4±0.7 |
| 5 | Serum amyloid P-component | APCS | Metabolism | P02743 | 25387/6.12 | 135 | 8 | ↓2.1±0.2 |
| 6 | Peroxiredoxin-1 | PRDX1 | Electron transport/Redox regulation | Q06830 | 22324/8.27 | 82 | 8 | ↓N/A |
| 7 | Creatine kinase B-type | CKB | Metabolism | P12277 | 42644/5.35 | 155 | 9 | ↓2.4±0.8 |
| 8 | Fibrinogen beta chain precursor | FGB | Metabolism | P02675 | 55928/7.95 | 179 | 16 | ↑2.6±0.9 |
| 9 | Cofilin-1 | CFL1 | [Structural](http://www.ebi.ac.uk/ego/DisplayGoTerm?id=GO:0007266) component | P23528 | 18371/8.26 | 280 | 15 | ↑3.7±0.9 |
| 10 | Hemoglobin subunit beta | HBB | Metabolism | P68871 | 15998/6.81 | 650 | 37 | ↓2.8±0.6 |
| 11 | NADH dehydrogenase flavoprotein 2 | NDUFV2 | Electron transport/Redox regulation | P19404 | 27392/5.71 | 148 | 5 | ↓2.2±0.4 |
| 12 | Fibrinogen gamma chain | FGG | Metabolism | P02679 | 51512/5.24 | 107 | 13 | ↑2.7±1.1 |
| 13 | Glyceraldehyde-3-phosphate dehydrogenase | GAPDH | Metabolism | P04406 | 35922/8.58 | 357 | 10 | ↑2.3±0.7 |
| 14 | Voltage-dependent anion-selective channel protein 1 | VDAC1 | Ion channel | P21796 | 30773/8.63 | 24 | 2 | ↑N/A |
| 15 | Fructose-bisphosphate aldolase A | ALDOA | Metabolism | P04075 | 39288/8.39 | 163 | 9 | ↑2.3±0.5 |
| 16 | Complement factor H-related protein 1 | CFHR1 | immune response | Q03591 | 35738/5.80 | 254 | 10 | ↓2.0±0.2 |
| 17 | DNA-binding protein Ikaros | IKZF1 | Zinc ion binding | Q13422 | 57528/6.12 | 178 | 17 | ↑2.1±0.4 |
| 18 | Coagulation factor VII | F7 | Metabolism | P08709 | 51594/7.73 | 165 | 9 | ↑3.7±0.8 |
| 19 | Glutathione S-transferase A1 | GSTA1 | Electron transport/Redox regulation | P08263 | 25672/8.91 | 240 | 4 | ↑2.4±0.9 |
| 20 | UMP-CMP kinase | CMPK1 | Metabolism | P30085 | 22222/5.44 | 233 | 7 | ↓N/A |
| 21 | Glutathione S-transferase P1 | GSTP1 | Electron transport/Redox regulation | P30042 | 25672/8.32 | 241 | 5 | ↓2.5±0.5 |
| 22 | Proteasome subunit beta type 2 | PSMB2 | Proteolysis | Q9NS71 | 22993/6.51 | 92 | 2 | ↓N/A |
| 23 | Carbonyl reductase [NADPH] 1 | CBR1 | Electron transport/Redox regulation | P16152 | 30641/8.55 | 327 | 11 | ↓2.7±1.1 |
| 24 | Ig kappa chain C region | IGKC | immune response | P01834 | 11773/5.58 | 459 | 12 | ↑2.3±0.6 |
| 25 | Cytochrome c oxidase subunit 5A,mitochondrial | COX5A | Electron transport/Redox regulation | P20674 | 16935/6.3 | 87 | 2 | ↓N/A |
| 26 | Fumarate hydratase, mitochondrial | FH | Tumor suppressor | P07954 | 54773/8.85 | 106 | 2 | ↓3.4±1.2 |
| 27 | Alpha crystallin B chain | CRYAB | [Apoptosis regulation](http://www.ebi.ac.uk/ego/DisplayGoTerm?id=GO:0006916) | P02511 | 20146/6.76 | 448 | 24 | ↓N/A |
| 28 | Pyruvate kinase isozymes M2 | PKM2 | Metabolism | P14618 | 57805/7.95 | 250 | 18 | ↑2.9±0.6 |
| 29 | Elongation factor Tu, mitochondrial | TUFM | Metabolism | P49411 | 49858/7.26 | 61 | 3 | ↑N/A |
| 30 | LIM and SH3 domain protein 1 | LASP1 | Zinc ion binding | Q14847 | 30097/6.61 | 84 | 4 | ↓3.4±1.2 |
| 31 | Ubiquitin-conjugating enzyme E2 N | UBE2N | Proteolysis | P61088 | 17184/6.13 | 91 | 2 | ↓3.1±0.7 |
| 32 | Nucleoside diphosphate kinase A | NME1 | Cell proliferation/differentiation | P15531 | 17309/5.83 | 125 | 11 | ↓2.2±0.3 |

All protein spots identified by ESI-Q-TOF MS/MS as significantly changed in average expression level in human lung adenocarcinoma compared with paired surrounding normal tissue. Lung adenocarcinoma tissues and corresponding normal tissues were collected from 9 lung adenocarcinoma patients.

^a^ Theoretical Mr/pI: Theoretical molecular weight (kDa) and pI from the ExPASy database.

^b^ No. of pep: Number of unique peptides identified by MS/MS sequencing.

^c^ Upward arrows: Up-regulated.

^c^ Downward arrows: Down-regulated.

^c^ N/A: the spots on one of the paired gels were too weak or nondetectable.
